# When the doorbell rings in COVID-19 times: Numerical insights into some possible scenarios

**DOI:** 10.1063/5.0045289

**Published:** 2021-04-28

**Authors:** Nirvik Sen, K. K. Singh

**Affiliations:** 1 Chemical Engineering Division, Bhabha Atomic Research Centre, Trombay, Mumbai 400085, India; 2 Homi Bhabha National Institute, Mumbai 400094, India

## Abstract

As ongoing Corona virus disease 2019 pandemic is ravaging the world, more and more people are following social distancing norms, avoiding unnecessary outings and preferring online shopping from the safety of their home over visiting brick and mortar stores and neighborhood shops. Although this has led to a significant reduction in chances of exposure, human-to-human interaction at the doorstep of the customer might be involved during the delivery of the ordered items. This human-to-human doorstep interaction arises in some other situations also. There is a finite probability that the person standing in front of the door coughs or sneezes during such an interaction. In this work, a three dimensional (3D) Euler–Lagrangian computational fluid dynamic model is used to understand the transmission and evaporation of micrometer-size droplets generated due to a coughing event in this setting. Different possible scenarios varying in wind direction, wind velocity, ventilation in the vicinity of door, and extent of door opening have been postulated and simulated. The results obtained from numerical simulations show that in the presence of wind, the dynamics of transmission of droplets is much faster than the dynamics of their evaporation. Thus wind velocity and direction have a significant impact on the fate of the droplets. The simulation results show that even if the door is opened by a very small degree, cough droplets enter through the door. Having open windows in the vicinity of the door on a windy day is expected to reduce the chance of the exposure significantly.

## I. INTRODUCTION

Corona virus disease 2019 (COVID-19) pandemic is one of the worst crises the modern world has faced. As of today (January 17th, 2021) more than 95 × 10^6^ cases of COVID-19 with about 2 × 10^6^ fatalities have been reported worldwide (Worldometer, 2021). The World Health Organization declared COVID-19 a Public Health Emergency of International Concern (PHEIC) on January 30, 2020 and a global pandemic on March 11, 2020 (Archived: WHO Timeline—COVID-19, 2020). About 10 months have passed since COVID-19 was declared a global pandemic, still at a global level it shows no sign of abating with the slope of the graph of cumulative COVID-19 cases vs time still increasing (Worldometer, 2021). Although COVID-19 vaccines have been developed and have gotten approvals for emergency use in several countries, social distancing is recommended as the best option to keep COVID-19 at bay (MOHFW, GOI, 2020; Oneindia News, 2020; Centers for Disease Control and Prevention, 2020). As more and more people are opting for social distancing, online shopping has seen a surge (The Washington Post, 2020; The Week, 2020). The e-commerce companies have developed and implemented Standard Operating Procedures (SOPs) for contactless deliveries (bigbasket.com, 2020; Business News, 2020). In the contactless delivery, the delivery person keeps the prepackaged stuff at the doorstep or, in the case of loose items, keeps them in the boxes/bags kept by the customer at the doorstep. The delivery person then rings the doorbell and goes back. However, there are many situations when one has to open the door when the doorbell rings. Some of these circumstances which are valid at least in Indian context are as follows:

(a) Delivery of liquid petroleum gas (LPG) cylinders in which one has to hand over the empty LPG cylinder to the delivery person and take from him the filled one in exchange. Payment is most often in cash. The whole process takes some time (at least 5 min).
(b) On the expiry of the credit card, the bank sends a new credit card through a courier service. The delivery person will ring the doorbell and will check the identity proof before handing over the package. Once again there will be a direct contact for some time.
(c) Several persons such as a milk vendor, a cable TV service provider, and a newspaper vendor will come to settle the monthly bill. Although digital transactions are on the rise, cash is still king in many countries including India. Thus the settlement process involving cash payment takes some time.

Despite widespread awareness related to COVID-19, there is a finite probability that the person standing at the doorstep with whom one interacts may not have a mask or the mask is not worn properly (Forbes, 2020; New York Times, 2020; and The Economic Times, 2020). On top of this finite probability is another finite probability of coughing or sneezing by the person with whom one interacts. Although the overall probability of this incidence is less, it is nevertheless finite. In this work we analyze by using numerical simulations what happens when such an event which, despite having low probability, does occur.

The physics involved is basically transmission and evaporation of micrometer-size droplets released from the mouth when a person coughs. The released droplets tend to fall to the ground while evaporation from them takes place. The distance covered by exhaled cough droplets and time taken by them to evaporate depend on climatic conditions (relative humidity and temperature) as well as aerodynamics prevailing at the instant of the coughing action (Mittal *et al.*, 2020). The problem of transmission and evaporation of micrometer-size droplets in different settings has been studied in several previously reported works. Ji and co-workers carried out numerical simulations to understand transmission and evaporation of micrometer-size droplets generated due to the coughing action inside a room having mixing or displacement type of ventilation (Ji *et al.*, 2018). Yang and co-workers used numerical simulations to understand the transmission and evaporation of micrometer-size droplets in a coach bus (Yang *et al.*, 2020). Feng and co-workers performed numerical simulations to understand how cough droplets spread form one person to the other under varying ambient conditions (Feng *et al.*, 2020). Dbouk and Drikakis used numerical simulations to understand the transmission and evaporation of droplets generated due to the coughing action in an open environment for varying wind conditions and relative humidity (Dbouk and Drikakis, 2020a). Li and co-workers studied the transmission and evaporation of cough droplets in heterogeneous humidity field produced as a result of mixing of supersaturated exhaled air and quiescent humid air (Li *et al.*, 2018). Yan and co-workers studied transmission and evaporation of cough droplets in a heterogeneous humidity field in the flow generated by the thermal effect of the body of a sitting person in an otherwise quiescent environment in an enclosed space (Yan and Li, 2019). Dbouk and Drikakis studied by using numerical simulations the phenomenon of transmission and evaporation of droplets generated due to the coughing action by a person wearing a face mask (Dbouk and Drikakis, 2020b). Risk of a virus laden particle transmission during flushing of urinal was also reported (Wang *et al.*, 2020). Recently computational fluid dynamic (CFD) based techniques have been used to track the transmission of virus laden droplets/particles inside an urban bus (Zhang *et al.*, 2021) and an elevator (Dbouk and Drikakis, 2021) in a classroom (Foster and Kinzel, 2021) or a cafeteria (Wu *et al.*, 2021).

Recently studies on estimation of drying time (diffusion limited evaporation) of cough/respiratory droplets (virus laden) falling on exposed surfaces have been reported (Bhardwaj and Agarwal, 2020). The effect of surface wettability on drying/evaporation time of droplets has also been studied using a novel mechanistic approach (Bhardwaj and Agarwal, 2020a). Recently a study revealed how virus (in a cough/respiratory droplet) can survive on the surfaces for days (Bhardwaj and Agarwal, 2020b). A correlation for unsteady evaporation of virus laden saliva droplets embedded in a CFD framework was recently used to estimate the effect of weather conditions on corona virus transmission (Dbouk and Drikakis, 2020c). Studies on application of numerical techniques to quantitatively determine efficacies of using face masks/shields amidst the pandemic have also been reported (Akhtar *et al.*, 2020; Verma *et al.*, 2020a; 2020b).

The main takeaway from the above-mentioned studies is that the governing equations which are used for modeling the phenomena of transmission and evaporation of micrometer-size droplets are fairly well known and have been applied to study transmission and evaporation of droplets in different settings and circumstances. In this study, we use the similar governing equations in a different setting.

Although the transmission and evaporation of cough droplets appear to be simple phenomena, actually these are very complex. Droplets released by coughing and sneezing may have widely varying size distributions (Han and Weng, 2013; Liu *et al.*, 2017). The angle at which the droplets are released during cough exhalation also affects the transmission of the droplets. The turbulent puff associated with coughing also affects trajectories of released cough droplets especially in closed environment. Ambient conditions such as temperature, relative humidity, and wind conditions prevailing at the instant of coughing also significantly affect the transmission and evaporation of droplets. Thus, each event of exhalation of cough droplets and their subsequent transmission and evaporation is unique and needs separate analysis.

The complexity of the phenomena often leads to invocation of some assumptions to get a working numerical model which gives reasonably accurate results with reasonable computational efforts. The model used in this study is premised on the assumptions that the cough droplets are ejected horizontally, the initial size distribution of the cough droplets follows Weibull probability density function, and the constituent of the cough droplets is water. These assumptions are similar to the ones invoked in several recent studies on transmission and evaporation of cough droplets (Dbouk and Drikakis, 2020b; 2020a). Since we have studied the transmission and evaporation in the presence of continuously blowing wind, the effect of very short-lived turbulent puff and plume of saturated exhaled air which accompanies exhalation of cough droplets is ignored. As discussed later, the suitability of this assumption is substantiated by a simulation carried out by considering turbulent puff also. The numerical model using the above-mentioned assumptions is implemented to study different possible scenarios involving a coughing event by a person standing in front of an open or partially open door of a flat for different wind conditions and ventilation in the vicinity of the door.

## II. NUMERICAL MODEL

### A. Computational domain

**FIG. 1.**
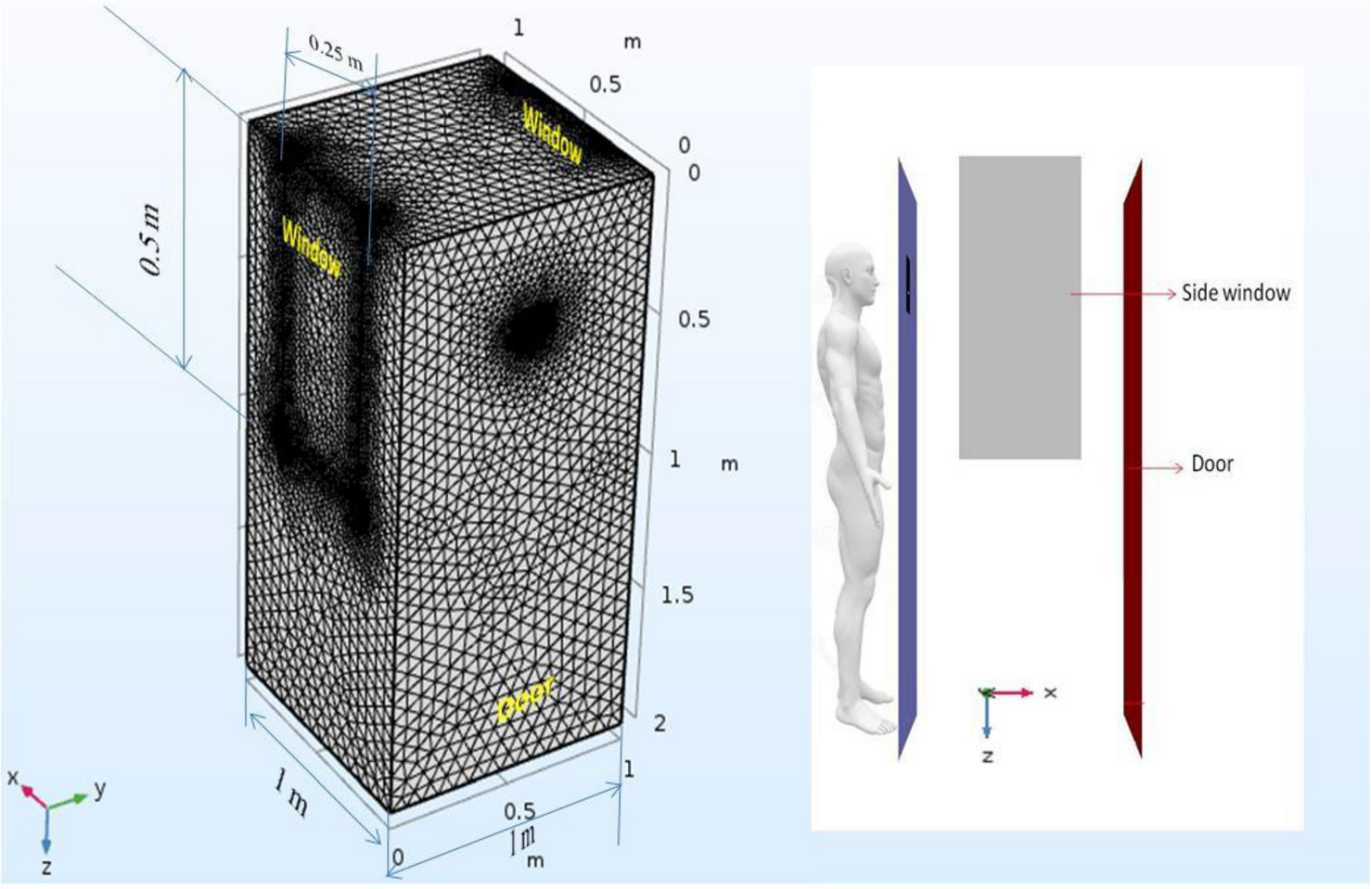
A typical computational domain used in the numerical simulations. The domain shown corresponds to a closed door.

A typical computational domain used in the numerical analysis is shown in Fig. 1. The dimensions of the door and other relevant dimensions used in the computational domain are as typically found in real life settings. The computational domain represents a person standing at some distance from the door which might be either partially open or fully open. Different scenarios have been simulated to understand the transmission and evaporation of droplets generated due to the coughing action by the person. The micrometer-size droplets generated due to the coughing action would travel through the domain. The path followed by the droplets will be significantly affected by the prevailing aerodynamics which will depend on the wind velocity and direction, and the ventilation conditions in the vicinity of the door. In case the door is partially open or fully open, the droplets might enter inside exposing the person interacting with the person standing in front of the door. The droplets will also undergo evaporation as they are transmitted. The evaporation rate will depend on the temperature and relative humidity in addition to its dependence on relative velocity between droplets and air. Some of the droplets may also undergo complete evaporation. In some cases, the droplets may be carried away by the wind without causing any exposure.

Unstructured tetrahedral meshing is used. An overall grid density of 2.90 × 10^5^ cells/m^3^ has been used. A non-uniform mesh is used. The regions near the nose and mouth of the person coughing are meshed using a refined grid, element size being 0.5 mm. For the remainder of the computational domain, a relatively coarser grid with an element size of 25 mm is used. The refinement is also carried out in the regions near the windows.

### B. Governing equations

An Euler–Lagrangian method is used for modeling. In this method the flow field of air is solved in the Eulerian frame of reference. The evaporation of cough droplets (modeled as discrete phase) depends on the temperature and relative humidity of the air. Turbulence in the air has been modeled by using a standard *k-ε* model. The governing equations solved for the air (continuous phase) are summarized in Table I.

**TABLE I.** The governing equations solved for the continuous phase (air).

$\nabla .\vec{u}=0$	Continuity equation
$\rho_{a}\left(\vec{u}.\nabla \right)\vec{u}=\nabla .\left[-p\overset{̿}{I}+\overset{̿}{\tau }\right]+\rho_{a}\vec{g}$	Momentum equation
$\overset{̿}{\tau }=\left(\mu +\mu_{T}\right)\left(\nabla \vec{u}+{\left(\nabla \vec{u}\right)}^{T}\right)$	Closure equation for the stress term in the momentum equation
$\rho_{a}\left(\vec{u}.\nabla k\right)=\nabla .\left(\frac{\mu_{T}}{\sigma_{k}}\nabla k\right)+G_{k}-\rho_{a}\varepsilon$	Conservation of turbulent kinetic energy
$\rho_{a}\left(\vec{u}.\nabla \varepsilon \right)=\nabla .\left(\frac{\mu_{T}}{\sigma_{\varepsilon }}\nabla \varepsilon \right)+\frac{\varepsilon }{k}\left(C_{1\varepsilon }G_{k}-C_{2\varepsilon }\rho_{a}\varepsilon \right)$	Conservation of turbulent energy dissipation rate
$\mu_{T}=\rho_{a}C_{\mu }\frac{k^{2}}{\varepsilon }$	Expression of turbulent viscosity
$G_{k}=\mu_{T}\left(\nabla \vec{u}+{\left(\nabla \vec{u}\right)}^{T}\right):\nabla \vec{u}$	Generation of turbulent kinetic energy

The cough droplets are treated as discrete particles in the Lagrangian frame. The transmission of the droplets depends on the flow field of air. The droplets undergo evaporation as they move. Due to evaporation, the size of droplets reduces with time. At the instant of their ejection from the mouth, the temperature of droplets would be close to the body temperature of a human being. Thereafter, the droplets would exchange heat with the surrounding air. The latent heat required for evaporation would result in reduction of the temperature of droplets which will continue to fall until the wet bulb temperature is reached. Thereafter, the sensible heat transfer from the ambient air to the droplets would provide the latent heat required for evaporation. The driving force for the evaporative mass transport would be the difference of the water vapor pressure at the surface of the drop and the partial pressure of water vapor present in the air. The equations which describe the motion and the evaporation of droplets are summarized in Table II. Drag force and gravitational force are considered acting on the droplets. The drag force exerted by the air on droplets has been modeled by using Schiller–Naumann drag model.

**TABLE II.** The governing equations solved for the discrete phase (droplets).

$m_{d}\frac{d\vec{u_{d}}}{dt}=\left(\rho_{d}-\rho_{a}\right)\vec{g}+\frac{C_{D}\pi d_{d}^{2}\rho_{a}}{8}\left|\vec{u_{d}}\left.-\vec{u}\right|\left(\vec{u_{d}}-\vec{u}\right)\right.$	Equation of motion of droplets
$C_{d}=\text{max}\left\{\frac{24}{{Re}_{d}}\left(1+0.15{Re}_{d}^{0.687}\right);0.44\right\}$	Drag model
$\frac{dm_{d}}{dt}=\dot{R}$	Rate of change of drop diameter with time
$\dot{R}=-\frac{2\pi d_{d}\;M_{w}D_{wv}}{\;R\;T}\Delta p\left(1+0.276\;{Re}_{d}^{1/2}{Sc}^{1/3}\right)$	Expression for accretion rate
${Re}_{d}=\frac{d_{d}\left|\vec{u_{d}}\right|\rho_{a}}{\mu_{a}}$	Drop Reynolds number
$Sc=\frac{\mu_{a}}{\rho_{a}D_{WV}}$	Schmidt number
$\Delta p=P_{T_{d}}^{s}-R_{H}P_{T}^{s}$	Equation for driving force for mass transfer
$\frac{dT_{d}}{dt}=\frac{3\lambda_{w}}{C_{pw}{\;d}_{d}}\frac{dd_{d}}{dt}+\frac{6h}{d_{d}\rho_{d}C_{pw}}\left(T-T_{d}\right)$ $\forall \;T_{wb}\leq T_{d}<T$	Rate of change of drop temperature with time
$Nu=\frac{h\;d_{d}}{k_{a}}=2+0.06\;{Pr}^{1/3}{Re}_{d}^{1/2}$	Correlation for Nusselt number
$Pr=\frac{\mu_{a}C_{pa}}{k_{a}}$	Prandtl number

### C. Initial, boundary conditions and numerical approach

The door is always defined as wall. The opening formed due to partial or complete opening of the door is defined as the pressure outlet. Velocity inlet has been defined at the face through which the wind is entering in the computational domain. No slip condition is used at the walls, floor, and ceiling.

Mouth has been modeled as a rectangular slit of dimensions (40 mm × 0.8 mm) (Dbouk and Drikakis, 2020a). Two different wind velocities (7.7 and 20 kmph) are considered in the simulations. The exhalation of droplets is assumed to be horizontal with a velocity of 8.5 m/s. The initial droplet size distribution is defined by Weibull probability density function (mean droplet size of 80 *μ*m and n = 8) as given by Eq. (1). For numerical solutions, the drop size distribution is discretized in 10 classes. Total mass of exhaled droplets is 7.7 mg and exhalation occurs over a duration of 0.12 s. The initial droplet size distribution, droplet velocity, total mass of droplets exhaled, and duration of exhalation are the same as used in a recent study (Dbouk and Drikakis, 2020a).

$$f=\exp\left\{-{\left(\frac{d_{d}}{\bar{d_{d}}}\right)}^{n}\right\},$$ (1)

*d_d_* is the droplet diameter, $\bar{d_{d}}$ is the mean droplet size, and *n* is the fitting exponent.

Continuity, and momentum equations are solved in a steady state manner to obtain the spatial distribution of velocity of air in the computational domain. Turbulence due to the wind is captured using standard k-ε model. Coupling of the Navier–Stokes equation with the conservation equations for turbulence parameters (namely, k and ε) is through a turbulent viscosity term. Once velocity field of air is obtained, particle tracking (Lagrangian) simulations are carried out to determine how the cough droplets spread based on the established velocity field for given ambient temperature and relative humidity conditions. The droplets of pure water are considered and are released as a distributed source over the mouth (40 mm × 0.8 mm). In majority of simulations, one way coupling of the droplets is considered. In the simulation that is carried out to show the effect of turbulent puff of air that gushes out of the mouth along with droplets, complete unsteady tracking of the particles is implemented. Time interval for the unsteady simulation is kept at 0.01 s.

### D. Validation of the model

**FIG. 2.**
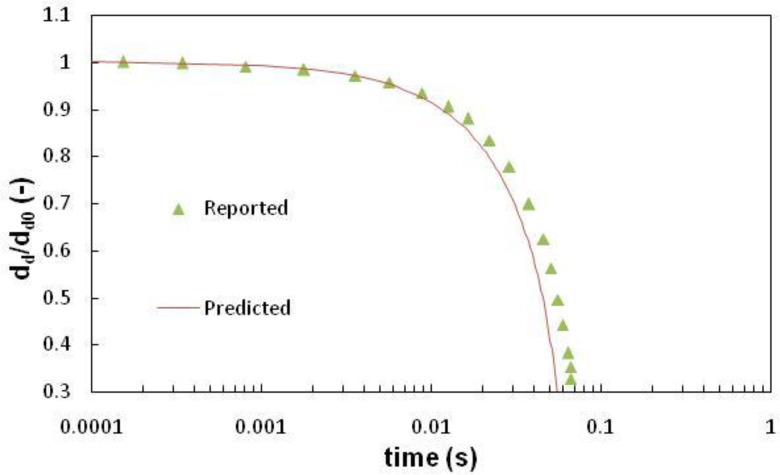
Comparison of CFD predicted drop size variation with time vis-a-vis results reported in the literature (d_d_ is the instantaneous droplet diameter while d_d0_ is the initial drop diameter).

The efficacy of the model used in this work is checked by modeling evaporation of a droplet in quiescent air. For this, a stationary droplet of initial diameter of 10 *μ*m evaporating in dry quiescent air having 25 °C dry bulb temperature is considered (Wei and Li, 2015). Figure 2 shows the comparison of the results predicted by the CFD model developed in this work with the results of the same case reported in the literature. A good match between the results predicted by our model with the results previously reported in the literature can be observed.

## III. RESULTS AND DISCUSSION

### A. Different postulated scenarios

The objective of this work is to simulate how cough droplets move in the vicinity of the door and to quantify the fraction of droplets entering through the open door. A variety of different scenarios have been simulated. The effects of the extent of door opening (30° degree open, 60° degree open, fully open), direction of wind (entering from the direction perpendicular to the closed door, entering from the right side window, entering from the left side window, and cross-flow), and wind velocity (7.7 and 20 kmph) have been studied. Table III summarizes all the different scenarios which have been simulated. In each scenario, the values tracked are the fractions of droplets evaporated, fallen on the floor or walls, fallen on the door surface, escaped (left the computational domain, except through the door), and entered through the open door. One of the many possible scenarios is the one in which there is no wind, the door is open, and the person standing in front of the door coughs or sneezes. In this case, due to the jet of the air that is exhaled along with cough or sneeze, droplets may be carried up to a distance of more than 1 m (Dbouk and Drikakis, 2020a). This will certainly expose the person standing at the door. This scenario with an obvious outcome is not simulated. In all the simulations, relative humidity and dry bulb temperature are considered to be 30% and 30 °C, respectively.

**TABLE III.** Different scenarios simulated.

Scenario	Purpose of simulation	Door	Wind direction	Wind velocity (kmph)	Left side window	Right side window
1	Effect of wind velocity	Fully open	Perpendicular to closed door	7.7	Closed	Closed
2				20		
3	Effect of extent of door opening	60° open	Perpendicular to closed door	20	Closed	Closed
4		30° open				
5	Effect of presence of windows	30° open	Perpendicular to closed door	20	Closed	Open
6					Open	Closed
7					Open	Open
8	Effect of wind direction for perpendicular entry of the wind from one of the windows	30° open	Perpendicularly from left side window	20	Open	Closed
9			Perpendicularly from right side window		Closed	Open
10			Perpendicularly from right side window		Open	Open
11	Effect of wind direction for oblique entry of the wind from one of the windows	30° open	Obliquely (30° toward door) from left side window	20	Open	Closed
12			Obliquely (60° toward door) from left side window		Open	Closed
13			Obliquely (30° toward door) from right side window		Closed	Open
14			Obliquely (30° toward door) from right side window		Open	Open

### B. Effect of wind velocity (scenarios 1 and 2)

Table IV summarizes the fate of the droplets for the scenarios 1 and 2 described in Table III. In these scenarios we consider the variation of wind velocity (wind coming from the direction perpendicular to the closed door). The scenarios pertain to two different wind velocities (7.7 and 20 kmph).

**TABLE IV.** Summary of the fate of the droplets exhaled in a cough event for different wind velocities in scenarios 1 and 2 (door fully open, wind coming in from the direction perpendicular to the closed door, and windows closed; RH = 30%, T = 30 °C).

Scenario	Fraction of droplets				
	Evaporated	Trapped	Trapped on door	Escaped	Entered through door
1 (V = 7.7 kmph)	0.2	0	0	0	0.8
2 (V = 20 kmph)	0.2	0	0	0	0.8

**FIG. 3.**
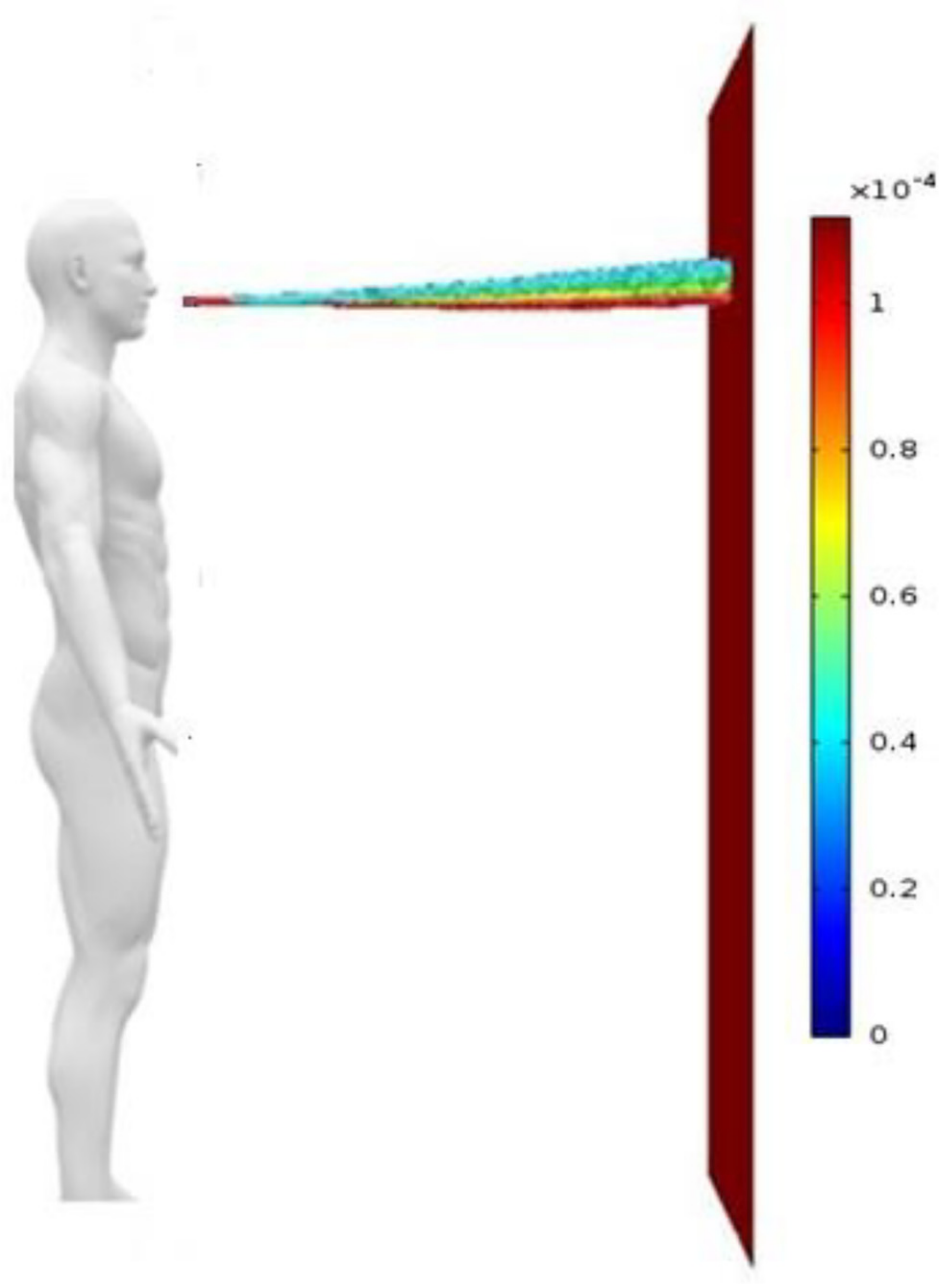
Side view of droplet tracks for scenerio-2 (wind blowing in the direction perpendicular to the closed door at a velocity of 20 kmph). The color scale is for drop diameter.

For both wind velocities, only 20% of the droplets get evaporated while traversing the distance between the source and the door. There is no difference in terms of fraction of droplets evaporated and fraction of droplets entering through the door if the wind velocity is raised from 7.7 kmph to 20 kmph. Hence, it can be observed that even a slight breeze is sufficient to carry the droplets through an open door in case the wind is coming in perpendicularly to the closed door. These results are at relatively low humidity and moderate ambient temperature at which the droplet evaporation rate is expected to be higher. At higher relative humidity and lower ambient temperatures, the fraction of droplets entering through the door will be even higher. Figure 3 shows droplets' tracks for wind velocity of 20 kmph. In the presence of the wind blowing in perpendicularly to the closed door, the droplets can be seen to enter the open door.

Typically coughing action in humans is associated with a turbulent puff of air along with exhaled droplets. Even though turbulent puff exists for a short duration of time, high velocity gradients created during coughing leads to significant turbulence which in turn leads to dispersal of exhaled droplets, especially finer droplets. The effect of this turbulent puff is very significant in closed environments (Li *et al.*, 2018). However, for the scenarios considered in this work, this is not the case. This is because the turbulence associated with the blowing wind tends to suppress that due to the coughing action. To substantiate this point, a simulation was carried out considering the effect of turbulent puff of air issuing out during the coughing action. In this case, the exhaled droplets were tracked in an unsteady manner along with the changes in the flow field due to coughing. Along with the droplets, air was injected into the computational domain (normal to mouth) at 8.5 m/s for a duration of 0.12 s (Dbouk and Drikakis, 2020a). The release of droplets (7.7 mg) was synchronized with the air puff ejection.

**FIG. 4.**
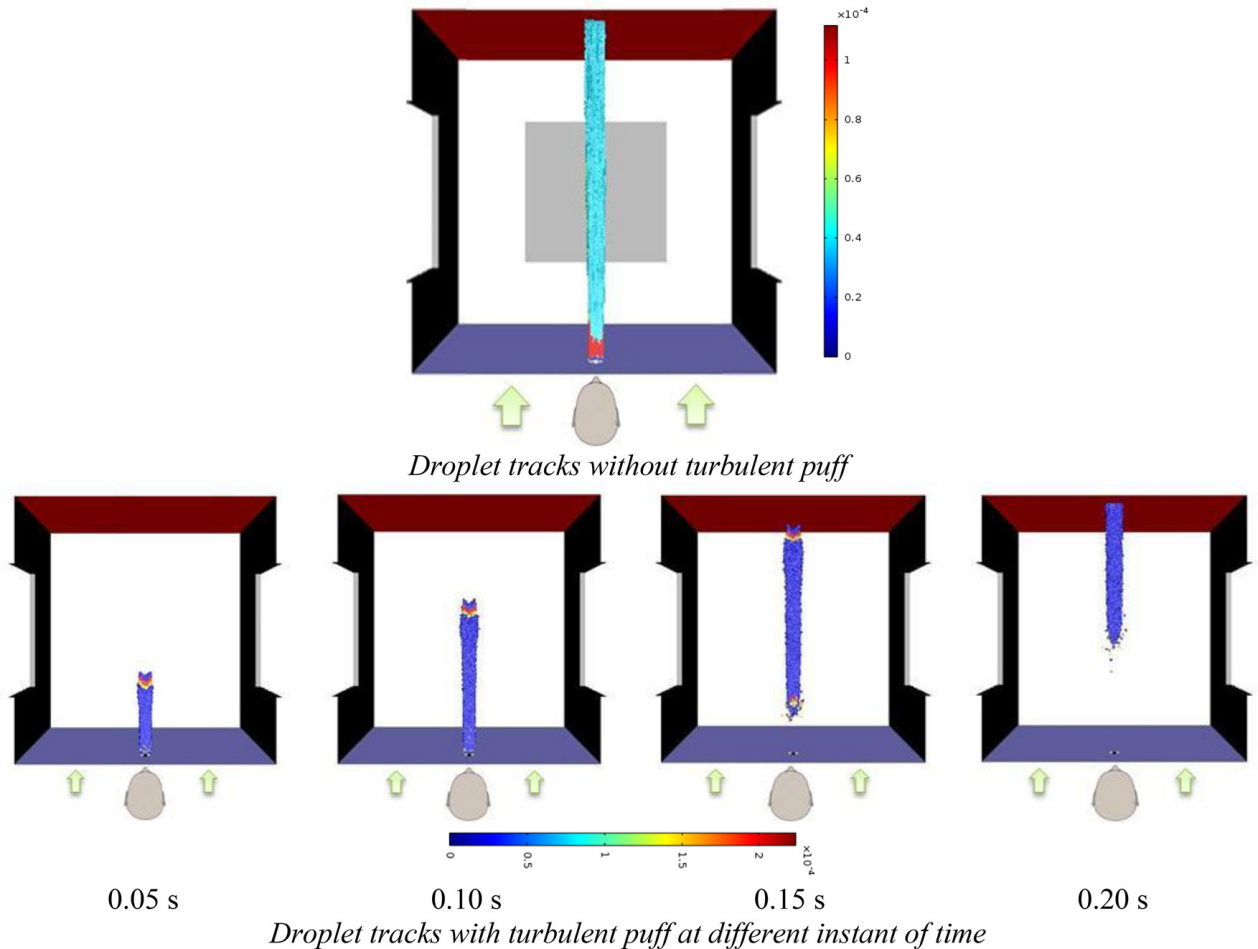
Droplet tracks without considering turbulent puff (top) and after considering turbulent puff (bottom) for the case of wind coming in the direction perpendicularly from the closed door. The door is fully open. The wind velocity is 20 kmph. Droplet tracks with turbulent puff are at different instant of time.

Figure 4 compares the results obtained from unsteady particle tracking simulations after incorporating the effect of turbulent air puff against that obtained from the case where turbulent air puff is not considered. It can be seen that the particle tracks are almost similar. In the presence of the wind coming in from the direction perpendicular to the closed door with a velocity of 20 kmph, the injected droplets are swept toward the door which is fully open without the formation of any cloud/plume which typically forms if someone coughs in a closed/isolated space (Li *et al.*, 2018). Thus, in the presence of the wind, the effect of turbulent puff associated with coughing is not significant. However, the computational time/resources necessary to carry out unsteady state simulations featuring turbulent air puff in a 3D domain are quite high. On the other hand, simulations without considering turbulent air puff can be done quickly without any appreciable effect on accuracy of results. Hence, for the rest of the work reported in this study, the later approach (not considering turbulent puff) has been followed.

### C. Effect of extent of door opening (scenarios 2–4)

Table V shows the fate of the droplets for the scenarios 2–4 mentioned in Table III. In this case, the fate of the droplets generated in the cough exhalation event is compared for three different extents of door opening. The door may be fully open or the door may be opened partially. Two different partial opening of door—30° and 60° degrees—are considered. A fully open door corresponds to 90° degree opening. Wind coming in from the direction perpendicular to the closed door with a velocity of 20 kmph has been considered in these simulations.

**TABLE V.** Summary of the fate of the droplets exhaled in a coughing event for different extent of door opening in scenarios 2–4 (wind coming in from the direction perpendicular to the closed door, V = 20 kmph, windows closed, RH = 30%, and T = 30 °C).

Scenario	Fraction of droplets				
	Evaporated	Trapped	Trapped on door	Escaped	Entered through door
2 (fully, i.e., 90° open door)	0.20	0	0	0	0.80
3 (60° open door)	0.20	0	0	0	0.80
4 (30° open door)	0.20	0	0	0	0.80

It can clearly be seen from Table V that for the case of the wind coming in from the direction perpendicular to the closed door with a velocity of 20 kmph, the fraction of droplets entering through the door is independent of the extent of door opening for door opening of 30° or more. In other words, even if the door is opened only by ∼30°, the situation would be as bad as opening the door completely. In these cases (door complete open, 60° open and 30° open) results show that 80% of cough droplets would enter through the door when relative humidity is 30% and ambient temperature is 30 °C.

**FIG. 5.**
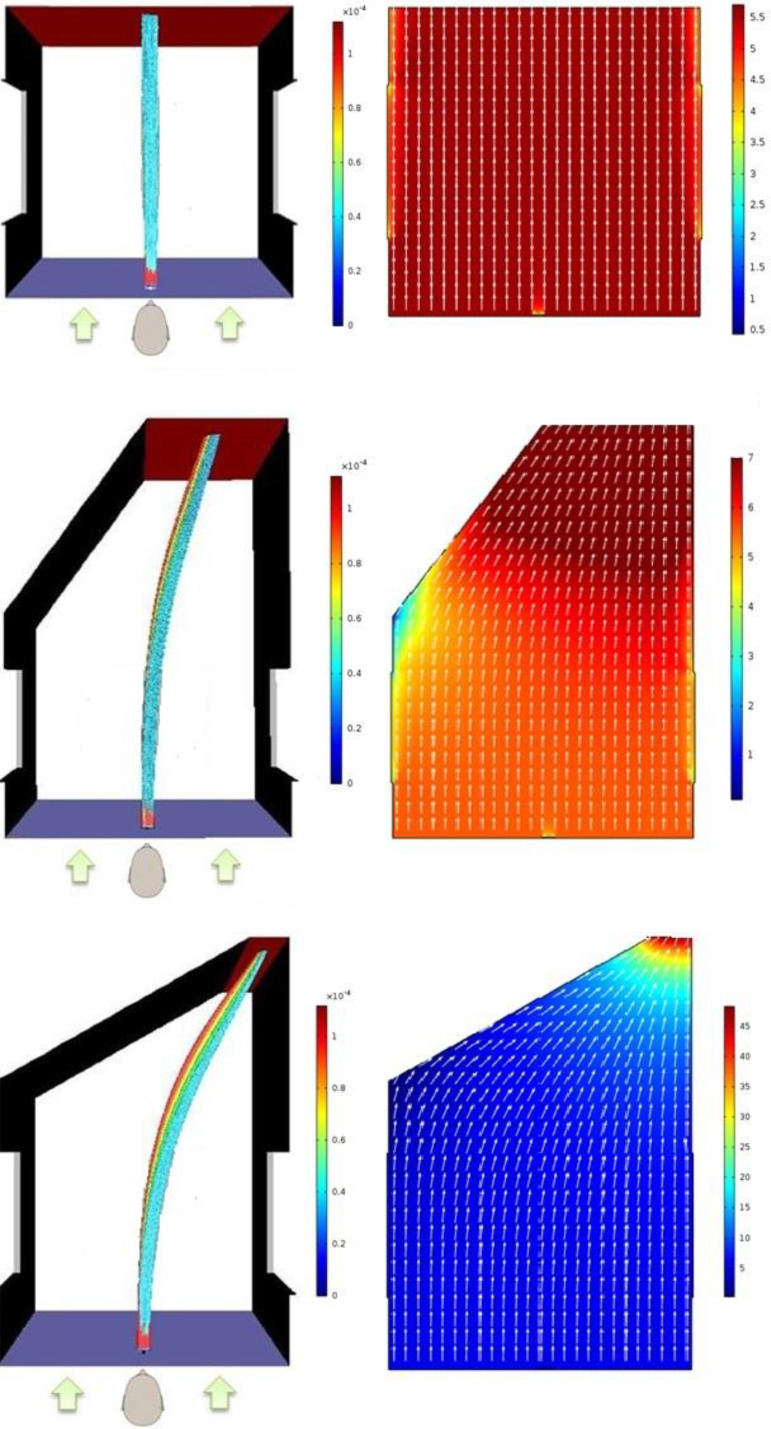
Droplets' tracks seen from the top (left) and velocity vectors and magnitude in a horizontal plane at the height of the mouth (right) for scenarios 2–4 varying in the extent of door opening. The arrows in the figures on the left side show the direction of wind. Scenerio-2 (top, door 90° open), scenerio-3 (middle, door 60° open), and scenerio-4 (bottom, door 30° open).

Conventional wisdom suggests that when the door is partially open, most of the droplets should come and stick to the door surface and only a small fraction of the exhaled droplets should enter inside through the door opening. However, simulation results indicate otherwise. Figure 5 shows top view of the droplets' tracks for all three cases mentioned in Table V. In addition, the velocity vectors of air in a horizontal plane at the height at which the droplets are exhaled are also shown alongside for each case. It can be seen that the droplets follow the velocity vectors of the air. This is attributed to the small size of the droplets because of which the drag force exerted on to the droplets by the wind is too strong to be overcome by the inertial force of or gravitational force acting on the droplets. One interesting observation in Fig. 5 for scenarios 3 and 4 is that the larger droplets tend to be on the left side as the smaller droplets tend to bend more as the droplets move along with the wind. This is because larger droplets, being heavier, have more inertia and align more slowly compared to the smaller droplets with the prevailing flow field of the wind. However, even the larger droplets are not too large to overcome the prevailing flow field of the wind to move straight.

**FIG. 6.**
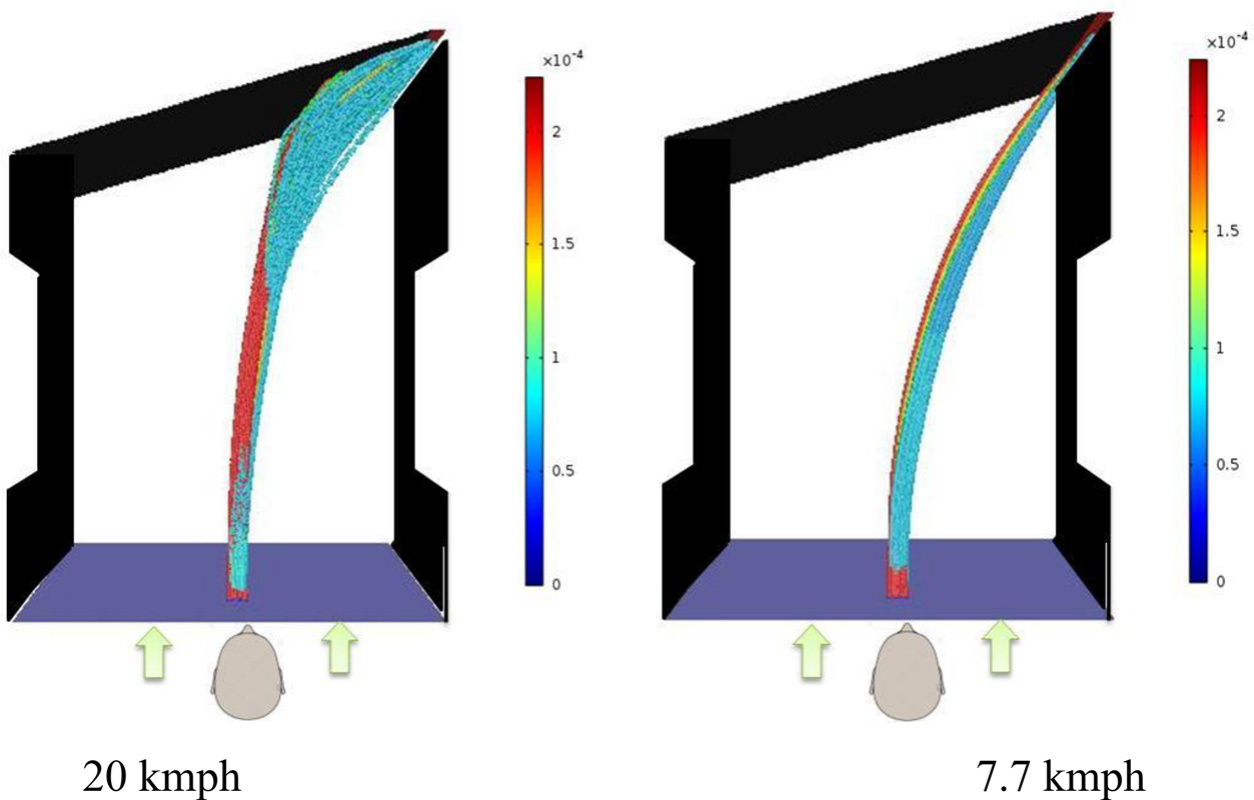
Comparison of droplets' tracks seen from the top for 15° door opening at two different wind velocities.

Thus it is seen that opening the door partially on a windy day represents the same risk as is associated with complete door opening. One interesting study will be to see what happens if the door opening is further reduced to 15° which translates to a gap of only 34 mm at the location of door opening. From a practical point of view, this corresponds to a situation where the door is opened slightly just to peep outside. Additional simulations are carried out at two different velocities (20 and 7.7 kmph). Figure 6 shows how the cough droplets are conveyed at two different velocities for the case of 15° door opening. For a flow velocity of 20 kmph, simulation results show that a fraction of the cough droplets hits the door and gets trapped on to the surface of the door (36.8%). However a majority of droplets still go through the gap (43.2%) created by the door opening. Hence even if the door is opened just for peeping outside (15°) in the presence of a strong wind, a significant fraction of cough droplets go through. The situation is even worse in case the wind velocity is on the lower side (7.7 kmph). In this case a greater fraction (80%) of the cough droplets enter through the door and no droplets are deposited onto the door surface. 20% of droplets evaporate for both cases. At higher velocity of 20 kmph, some of the larger sized droplets are dragged by the high velocity wind and are impacted onto the door. This phenomenon is observed only for the 15° door opening and not for other cases shown in Fig. 5 (for 20 kmph wind velocity). This is because relatively larger droplets do not get time to get completely aligned with the wind entering the door opening due to higher local velocities caused by a small door opening at a higher wind velocity. In fact the droplets gets only around 0.12 s before impacting the door. At low velocity the droplets get sufficient time (∼0.45 s) to align to the wind direction and go into the door opening. Thus simulation results clearly show that opening the door even for peeping outside even at a modest wind velocity may lead to exposure.

### D. Effect of presence of windows (scenarios 5–7)

**FIG. 7.**
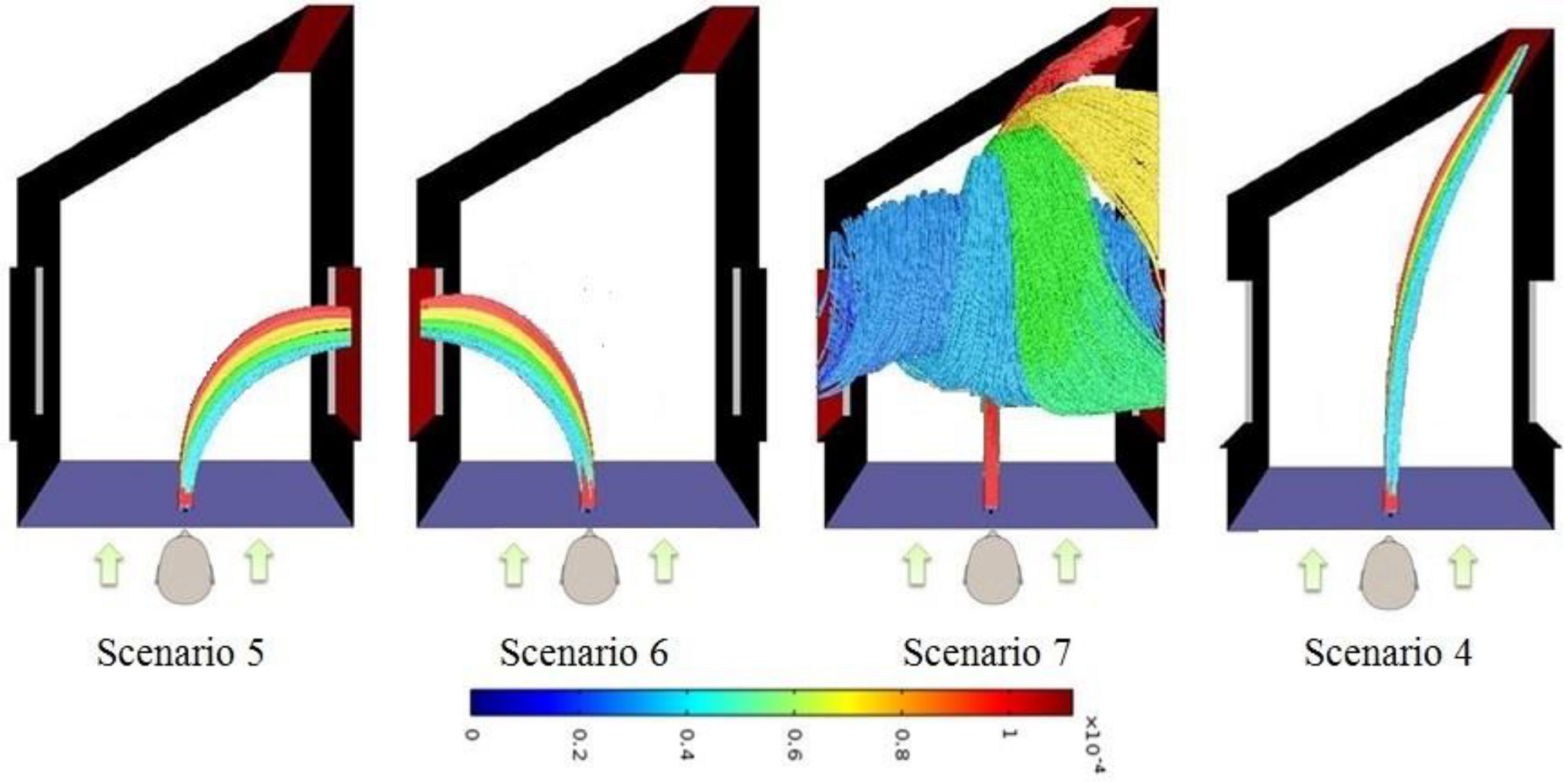
Droplets' tracks seen from the top for scenarios 4–7. The arrows show the direction of wind entry in the computational domain.

In Secs. III B and III C in which the effect of wind velocity and the effect of extent of door opening are discussed, it is assumed that the windows on the right side and the left side are closed and the wind is coming in from the direction perpendicular to the closed door. This situation is clearly the worst condition because the wind along with the droplets enter through the door. However, the situation would be different if the right and/or left window are open. Results of the simulations pertaining to scenarios 5–7 are presented in this section and compared against scenario 4 which corresponds to the case in which both windows are closed. Table VI summarizes the fate of the droplets for the scenarios 5–7 mentioned in Table III. Results of scenario 4 are included for sake of comparison. In these scenarios, wind velocity is kept constant at 20 kmph. Figure 7 shows the tracks of the droplets for scenarios 4–7.

**TABLE VI.** Summary of the fate of the droplets exhaled in coughing events described by scenarios 4–7 (wind coming in from the direction perpendicular to the closed door, V = 20 kmph, RH = 30%, T = 30 °C, and 30° door opening).

Scenario	Fraction of droplets				
	Evaporated	Trapped	Trapped on door	Escaped	Entered through door
5 (right side window open, left side window closed)	0.2	0	0	0.8	0
6 (right side window closed, left side window open)	0.2	0	0	0.8	0
7 (both right side and left side windows open)	0.224	0.405	0	0.354	0.017
4 (both right side and left side window closed)	0.2	0	0	0	0.8

As can be seen form Table VI and Fig. 7, in case the left side window is open and the window on the right side is closed, majority of the droplets exit the domain through the open window. Similarly, if the window on the right side is open and the window on the left side is closed, majority of the droplets exit the domain through the open window. Therefore, when either the window on the right side or the left side is open, no droplet enters the door opening. The droplets go out through the open window along with the wind which takes the path of the least resistance. However, when both the windows are open, there is significant churning of the droplets within the computational domain. In this case the incoming air will get three paths to flow through—through the right window, left window, and the open door. The churning in the computational domain may be attributed to the fact that the resistance offered by these three possible pathways is similar to each other and there is no preferential path of least resistance. Finally, the heavier cough droplets tend to follow on their course due to their inertia and enter the partially opened door while the smaller droplets get caught in the churn and exit through the windows. The results for scenarios 5–7 are totally different from that for scenario 4 in which 80% of the droplets go through the open door. Amongst scenarios 5–7, in the worst case (i.e., scenario 7) only 1.7% of the droplets enter the door. Thus it can be concluded that having the windows in the vicinity of door and keeping them open reduces the risk significantly on a windy day when the wind is blowing in from the direction opposite to the closed door.

**FIG. 8.**
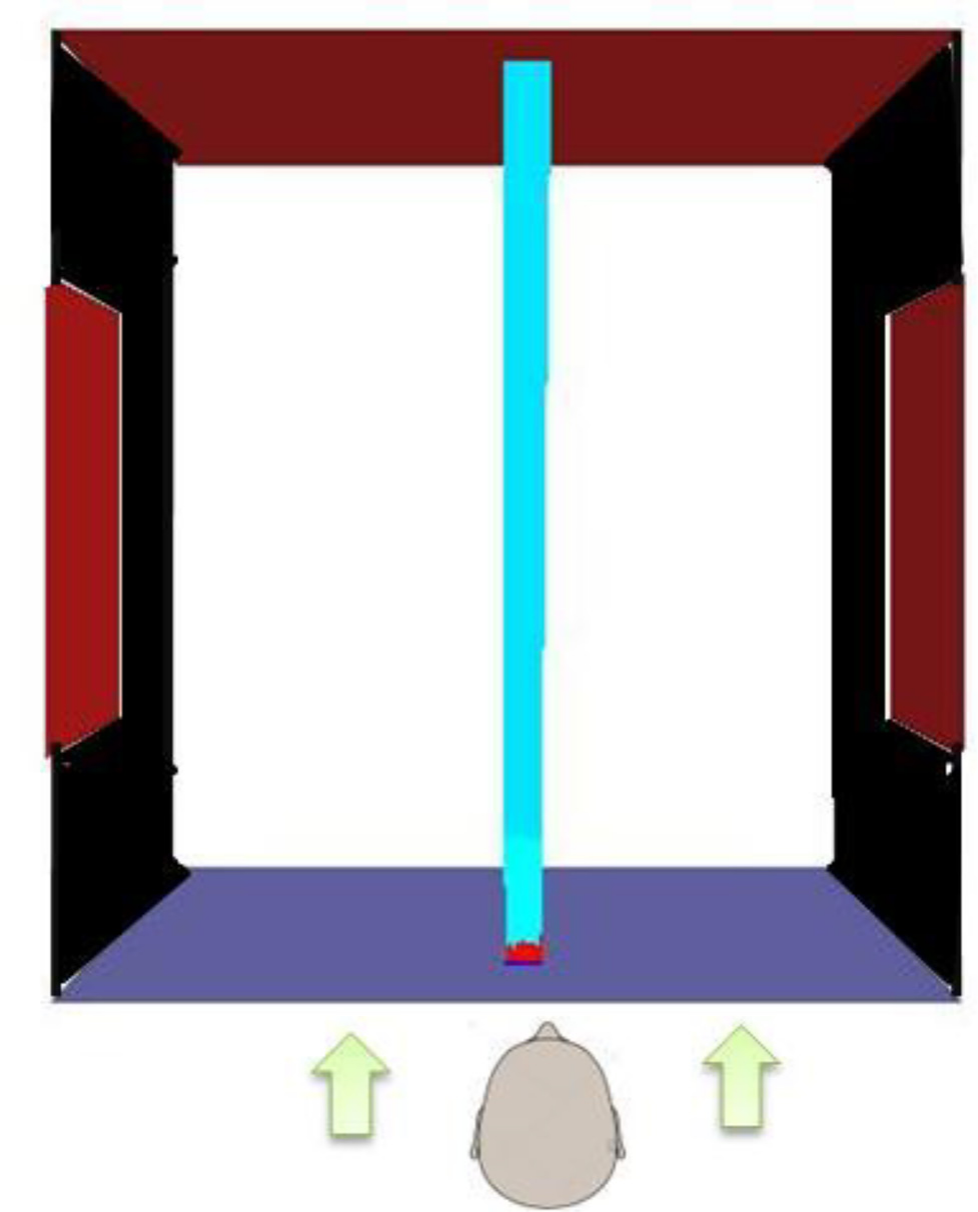
Droplets' tracks seen from the top for the case when both windows are open as well the door is completely open.

It may be noted that in case the extent of door opening is more than 30°, the aerodynamics will change. Figure 8 shows the situation when the door is completely open along with the two windows. In this case, the wind predominantly flows straight rather than exiting through the window(s) and thus the droplets are carried inside. In this case 80% of the cough droplets enter through the door while the remaining 20% gets evaporated. This is because, out of the three possible exits—straight through the door, through the right window, and through the left window, the wind carrying the droplets prefers the path of least resistance and continues its course straight toward and through the open door. In this case, the presence of windows does not matter and the case is just as bad as if there were no windows.

### E. Effect of direction of wind

#### 1. Perpendicular entry of the wind from one of the windows (scenarios 8–10)

Table VII summarizes the fate of the droplets for scenarios 8–10 defined in Table III. The results for scenario 4, discussed earlier, have also been included for comparison. In these scenarios, the effect of the direction of the wind on the fate of droplets is studied. Wind is assumed to be entering perpendicularly. In all the previous scenarios discussed so far, the wind was considered coming in from the direction perpendicular to the closed door. In scenarios 8 and 9, two more directions of wind entry have been considered—from the window on the left side and the window on the right side, respectively. In these two scenarios, only one of the windows is open and the other is closed. The cross-flow condition, in which both windows are open, is considered in scenario 10. In all the simulations, a partially open door (30° open) and wind velocity of 20 kmph is considered. In scenarios 8–10, the face opposite to the closed door is also considered as outflow signifying that a part of wind entering through the window may leave from this face of the computational domain.

**TABLE VII.** Summary of the fate of the droplets exhaled in a coughing event for perpendicular entry of the wind from different directions in scenarios 4, 8–10 (V = 20 kmph, door is 30° open, RH = 30%, T = 30 °C).

Scenario	Fraction of droplets				
	Evaporated	Trapped	Trapped on door	Escaped	Entered through door
4 (wind coming in from direction perpendicular to the closed door)	0.2	0	0	0	0.8
8 (wind from left window, right window closed)	0.4	0	0	0.6	0
9 (wind from right window, left window closed)	0.34	0.01	0	0.65	0
10 (wind from right window, left window open)	0.42	0.21	0	0.37	0

It can be seen that the wind direction has a very important role in determining the fraction of droplets that eventually enter through the door opening. It can be seen that when the wind is blowing in from the direction opposite to the closed door (scenario 4), majority of the droplets enter the room. However, when the wind is blowing from either the window on the right side or the left side, none of the droplets enter through the door opening and most of them escape through the open boundaries. This is the case for cross-flowing wind also.

Fraction of droplets evaporated is the maximum for the case of cross-flow condition (scenario 10). For all the cases in which wind enters perpendicularly, significant fraction of droplets escape the computational domain indicating that the droplets are swept away from the vicinity of the door without posing any risk. Clearly, having windows, which can facilitate perpendicular entry of the wind on a windy day, helps as this wind acts as a curtain for the droplets and prevent them from reaching the door.

**FIG. 9.**
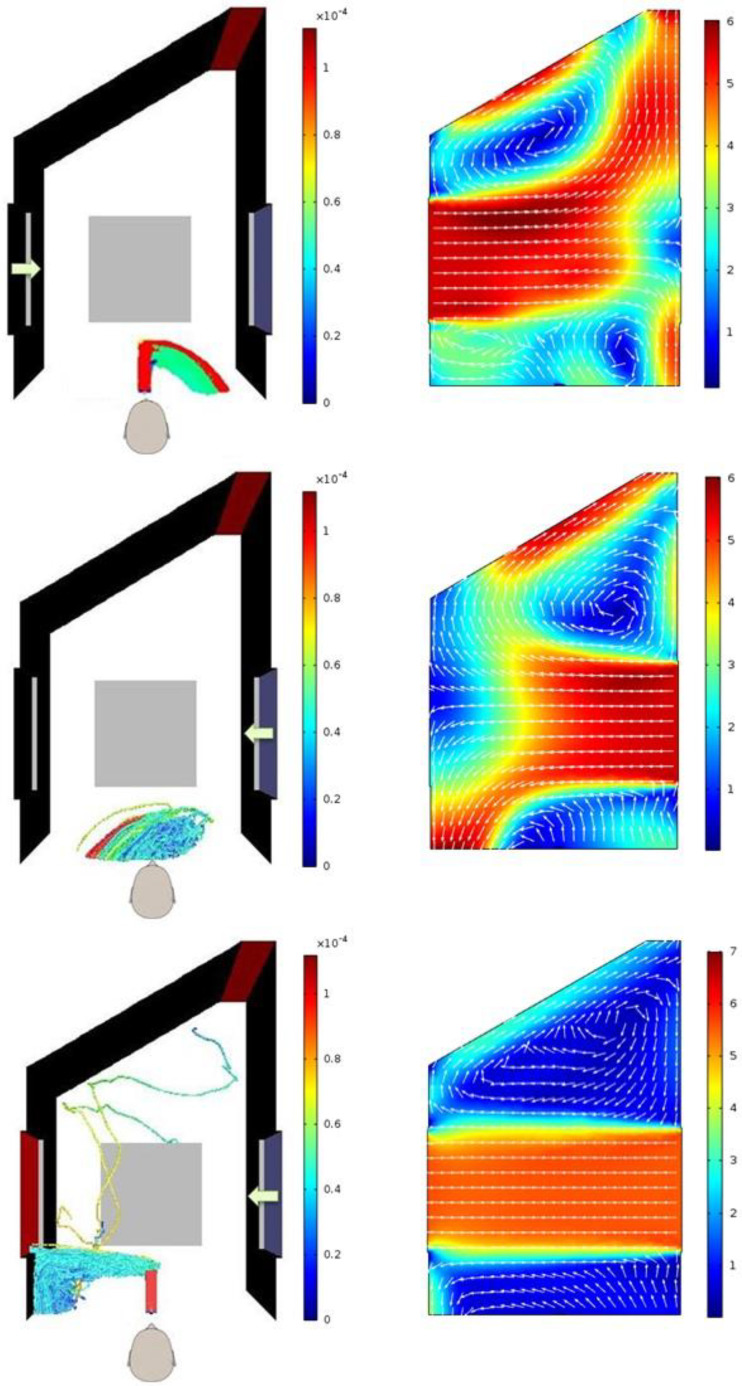
Top view of droplets' track (left) and velocity vectors in a horizontal plane at the height of mouth (right) for scenarios 8–10. The arrows in the figures on the left side show the direction of the wind entry. Top: Scenario-8 (wind in from the left window, the right window closed). Middle: Scenerio-9 (wind in from the right window, the left window closed). Bottom: Scenerio-10 (wind from the right window, the left window open).

Figure 9 shows the top view of the droplets' tracks along with the velocity vectors in a horizontal plain at the height of the mouth for scenarios 8–10. The droplets are found to follow the velocity vectors. When wind is blowing in from the left window (scenario 8 in Fig. 9) and the window on the right side is closed, the droplets get entrained in the dominant flow direction and escape the computational domain from the face opposite to the closed door. The wind enters the room but droplets do not. The bigger droplets are seen to be on the outer boundary of the cluster of the droplets escaping the domain (scenarios 8 and 9 in Fig. 9) due to their inertia. The presence of recirculatory flow of air is clearly visible for all the cases in which the wind enters from the left or the right side window.

For scenario-10 (cross-ventilation) most of the droplets get trapped in the recirculation and either hit the left wall of the computational domain or go out. Only a few droplets are observed to cross the high velocity gush of the cross-wind to make it toward the door. But by the time they reach the door they too evaporate.

#### 2. Oblique entry of the wind from one of the windows (scenarios 11–14)

**FIG. 10.**
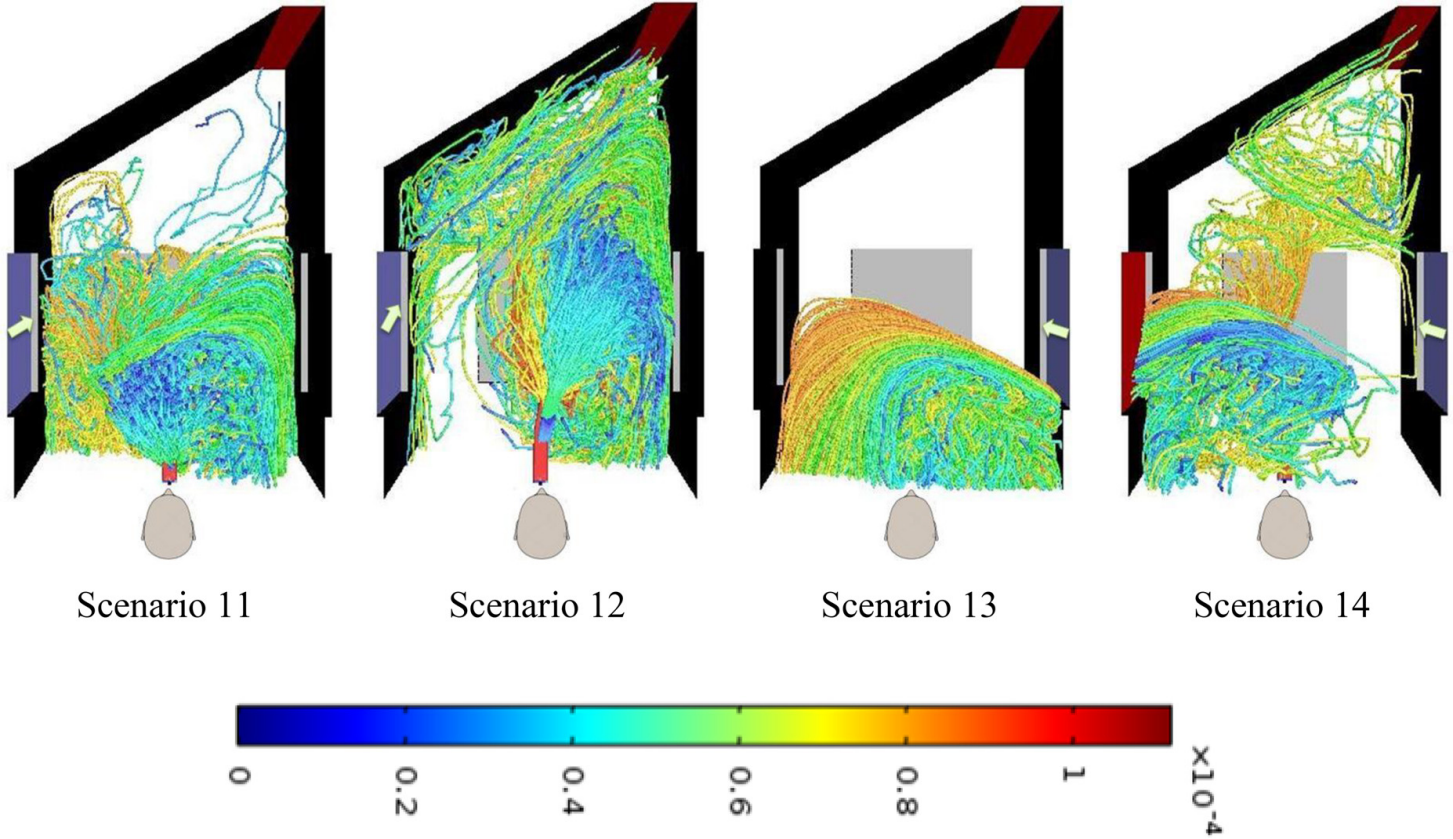
Top view of droplets' track (left) for scenarios 11–14. The arrows indicate the direction of the wind entry.

In Sec. III E 1 the wind blowing from the left/right window was entering perpendicularly. However, it is possible that the wind from the window does not enter perpendicularly but at an angle to the face representing the window. Results of a few such scenarios (11–14), detailed in Table III, are presented in this section. Table VIII summarizes the fate of the droplets for these scenarios. In these scenarios, the face opposite to the closed door too is considered as an outflow boundary signifying that a part of wind entering through the window may leave from this face also. Figure 10 shows the tracks of the cough droplets for these scenarios.

**TABLE VIII.** Summary of the fate of the droplets exhaled in the coughing event for oblique entry of the wind from different directions in scenarios 11–14 (V = 20 kmph, door is 30° open, RH = 30%, T = 30 °C).

Scenario	Fraction of droplets				
	Evaporated	Trapped	Trapped on door	Escaped	Entered through door
11 (oblique wind from left side window, 30° toward the door, right side window closed)	0.700	0.075	0	0.225	0.000 4
12 (oblique wind from left side window, 60° toward the door, right side window closed)	0.654	0.119	0.003	0.216	0.008
13 (oblique wind from right side window, 30° toward door, left side window closed)	0.222	0.013	0	0.765	0
14 (oblique wind from right side window, 30° toward door, left side window open)	0.605	0.075	0.001	0.315	0.004

It can clearly be seen from Fig. 10 that the fraction of droplets that enters the door opening is more when the wind enters through the left window at an angle of 60° toward the door (scenario 12). No droplet is seen to go through the door opening when the wind blows in at an angle of 30° from the right side window when the left side window is closed (scenario 13). We can compare scenario 14 with 10 in both of which the winds enter from the right side window with window on the left side open (cross-flow condition). When the wind blew perpendicular to the window under cross-flow conditions (scenario 10), no droplets would enter the door opening. However, if the wind is inclined toward the door (30°), around 0.4% of the droplets do enter the door opening.

Comparison of Tables VII and VIII shows that, while, unlike the perpendicular entry of the wind, oblique entry of the wind through the window may not completely prevent droplets from entering through the door opening, the fraction of droplets that may enter through door opening is very small. Thus having windows in the vicinity of the doors is definitely desirable.

Oblique entry of the wind from the left side window or the right side window at an angle pointing away from the door is not simulated. In these cases, the droplets will certainly be swept away from the door.

## IV. CONCLUSIONS

A 3D Euler–Lagrangian model is used to predict the consequences of a typical cough exhalation event during doorstep interaction of two human beings on a windy day. The exhaled droplets would move around and evaporate. Their transmission and evaporation rates depend on the prevailing velocity field, temperature, and humidity. Several scenarios have been postulated and evaluated. The effects of wind velocity, wind direction, and extent of door opening on fraction of droplets entering the door opening are quantified. Based on the results obtained in this work the following conclusions can be made:

(a) Even a light breeze coming in from the direction perpendicular to the closed door is sufficient to carry the droplets inside the room if the door is open and there are no windows in the vicinity of the door or the windows are closed. In this case, the extent of door opening does not matter. A fully open door or a partially opened door (opened to peep through) leads to the same result. In both cases a significant fraction of droplets may enter the room.
(b) However, if there are windows in the vicinity of door and either one or both of them are open, the fraction of droplets entering the room is significantly reduced when the wind is coming in perpendicularly to the closed door and door opening is small. In this case (windows present and at least one is open) the extent of door opening may matter. Small door opening is expected to give reduced exposure.
(c) Cross-ventilation in front of the door significantly reduces the possibility of droplets entering the room when the door is slightly open (∼30°). Even if there is a single window through which wind blows in perpendicularly, there is very less probability of the drops entering the room. The wind entering perpendicularly through the window acts as an air curtain preventing the droplets from entering the room. The air will go inside the room but drops would not be carried inside. However, the situation slightly worsens if the wind blowing from the window enters obliquely inclined toward the door. Still the fraction of droplets entering the room will remain small. In this case also, the extent of door opening would matter.

It may be noted that the scenarios evaluated here are just few of many possible scenarios. It is practically not possible to evaluate all scenarios. For examples, the local wind direction may not be steady, wind velocity may not be horizontal, the person might bend his head down while coughing leading to exhalation of cough droplets not in a horizontal plane but at an angle, aerodynamic will be quite complex when the event of door opening, and cough exhalation occurs at the same or almost at the same instant. The drop size distribution and the amount of cough droplets exhaled might also vary. Thus each event of cough exhalation is a unique event and the quantitative results may vary from event to event. Nevertheless, findings such as having windows in the vicinity of the door will reduce the risk of exposure on a windy day will hold in general. However, it is advised to continue to exercise utmost caution during human to human doorstep interactions even if the vicinity of door is well ventilated.

COVID-19 pandemic has taught us innumerable lessons, some are field specific and some are for the entire mankind (Fontana and Arkenau, 2020; Iyengar *et al.*, 2020; Lau *et al.*, 2020; and Megahed and Ghoneim, 2020). One of our takeaways is that the designs should not only be fool-proof but flu-proof also. We hope that the findings reported in this study will help in this endeavor.

## Data Availability

The data that support the findings of this study are available from the corresponding author upon reasonable request.

## NOMENCLATURE

*C_d_*: Drag coefficient (…)
*C_pa_*: Specific heat capacity of air (J kg^−1^ K^−1^)
*C_pw_*: Specific heat capacity of water (J kg^−1^ K^−1^)
*d_d_*: Droplet diameter (m)
*d_d0_*: Initial droplet diameter (m)
*D_wv_*: Diffusivity of water vapor in air (m^2^ s^−1^)
$\vec{g}$: Acceleration due to gravity (m s^−2^)
*G_k_*: Turbulent kinetic energy generation rate (kg m^−1^ s^−3^)
*h*: Heat transfer coefficient (W m^−2^ K^−1^)
*k*: Turbulent kinetic energy (m^2^ s^−2^)
*k_a_*: Thermal conductivity of air (W m^−1^ K^−1^)
*m_d_*: Mass of droplet (kg)
*Nu*: Nusslet number (…)
*Pr*: Prandlt number (…)
*p*: Static pressure (Pa)
$P_{T}^{s}$: Saturation pressure of water vapor at dry bulb temperature (Pa)
$P_{T_{d}}^{s}$: Saturation pressure of water vapor at droplet temperature (Pa)
*R*: Universal gas constant (J mol^−1^ K^−1^)
$\dot{R}$: Accretion rate (kg s^−1^)
*Re_d_*: Droplet Reynolds number (…)
*R_H_*: Relative humidity (…)
*Sc*: Schmidt number of air (…)
*T*: Temperature of air (K)
*T_d_*: Droplet temperature (K)
*T_wb_*: Wet bulb temperature (K)
$\vec{u}$: Velocity vector of air (m s^−1^)
${\vec{u}}_{d}$: Droplet velocity (m s^−1^)
*V*: Wind velocity (m s^−1^)

**Greek letters**
*Δp*: Driving force for mass transfer (Pa)
*ε*: Turbulent energy dissipation rate (m^2^ s^−3^)
*λ_w_*: Latent heat of water (J kg^−1^)
*μ*_*a*_: Viscosity of air (Pa s)
*μ_T_*: Turbulent viscosity (Pa s)
*ρ*_*a*_: Density of air (kg m^−3^)
*ρ_d_*: Droplet density (kg m^−3^)
$\overset{̿}{\tau }$: Total stress (Pa)
